# Genetic architecture of the response of *Arabidopsis thaliana* to a native plant-growth-promoting bacterial strain

**DOI:** 10.3389/fpls.2023.1266032

**Published:** 2023-11-09

**Authors:** Daniela Ramírez-Sánchez, Chrystel Gibelin-Viala, Fabrice Roux, Fabienne Vailleau

**Affiliations:** LIPME, INRAE, CNRS, Université de Toulouse, Castanet-Tolosan, France

**Keywords:** PGPB, *Pseudomonas siliginis*, seed inoculation, vegetative growth, negative tradeoff, GWA mapping

## Abstract

By improving plant nutrition and alleviating abiotic and biotic stresses, plant growth-promoting bacteria (PGPB) can help to develop eco-friendly and sustainable agricultural practices. Besides climatic conditions, soil conditions, and microbe-microbe interactions, the host genotype influences the effectiveness of PGPB. Yet, most GWAS conducted to characterize the genetic architecture of response to PGPB are based on non-native interactions between a host plant and PGPB strains isolated from the belowground compartment of other plants. In this study, a GWAS was set up under *in vitro* conditions to describe the genetic architecture of the response of *Arabidopsis thaliana* to the PGPB *Pseudomonas siliginis*, by inoculating seeds of 162 natural accessions from the southwest of France with one strain isolated from the leaf compartment in the same geographical region. Strong genetic variation of plant growth response to this native PGPB was observed at a regional scale, with the strain having a positive effect on the vegetative growth of small plants and a negative effect on the vegetative growth of large plants. The polygenic genetic architecture underlying this negative trade-off showed suggestive signatures of local adaptation. The main eco-evolutionary relevant candidate genes are involved in seed and root development.

## Introduction

Plant-Growth-Promoting Bacteria (PGPB) are bacterial strains isolated from diverse environmental reservoirs with the potential to provide multiple benefits to food and non-food crops (Bashan, 1998; Glick, 2012; Santoyo et al., 2016; Ramakrishna et al., 2019; Tian et al., 2020). For instance, PGPB can promote plant growth by improving plant nutrition and alleviating abiotic stresses such as drought and salinity (Choudhary et al., 2016; Singh et al., 2018; Kumar et al., 2020; Mokrani et al., 2020; Santoyo et al., 2021; Gamalero and Glick, 2022; Gupta et al., 2022). PGPB can also promote plant health by participating in defense against pathogens and pests (Liu et al., 2017; Liu et al., 2020; Morelli et al., 2020; Ruiu, 2020; Noman et al., 2021; Santoyo et al., 2021). In addition to the potential of PGPB to increase crop yield, PGPB can contribute to reducing environmental degradation by participating in phytoremediation techniques for soil and water decontamination (de-Bashan et al., 2012; Glick, 2012; Santoyo et al., 2016; Martínez-Hidalgo et al., 2019; Riva et al., 2020).

PGPB represent therefore a unique opportunity to develop eco-friendly and sustainable agricultural practices, a lofty goal that is especially relevant in the context of current global changes (Glick, 2012; Santoyo et al., 2016; Ramakrishna et al., 2019). For instance, such practices include the biotechnological use of PGPB as biofertilizers or biocontrol agents (De Silva et al., 2019; Leila and El-Hafid, 2020; Ünüvar and Ünlü, 2022). However, the effectiveness of most PGPB is highly influenced by climatic conditions, soil conditions, and microbe-microbe interactions, thereby deeply affecting their use in a wide range of agricultural conditions (Fageria and Stone, 2006; Gaiero et al., 2013; Rilling et al., 2019; Canfora et al., 2021). Moreover, the effects of a PGPB can highly depend on the genotype of the host plant (Wintermans et al., 2016; Ramakrishna et al., 2019; Yassue et al., 2021; Ramírez-Sánchez et al., 2022; Schultz et al., 2022). There is, therefore, a growing interest in the potential of harnessing the beneficial effects of individual members of the microbiota through plant breeding (Bergelson et al., 2021; Gutierrez and Grillo, 2022; Nerva et al., 2022; Santoyo, 2022). This requires the identification of host genetic factors either by using artificial genetic variation or by exploiting natural genetic variation (Bergelson et al., 2021). The latter approach was adopted by setting up genome-wide association studies (GWAS) on diverse plants including the model plants *Arabidopsis thaliana* (Wintermans et al., 2016; Cotta et al., 2020; Plucani do Amaral et al., 2023) and *Medicago truncatula* (Stanton-Geddes et al., 2013) as well as diverse crops such as maize (Vidotti et al., 2019; Yassue et al., 2021; Yassue et al., 2023), soybean (Torkamaneh et al., 2020) and common bean (Kamfwa et al., 2015). These GWAS revealed a highly polygenic architecture of response to PGPB, with the identification of multiple Quantitative Trait Loci (QTLs) with small effects. The fine mapping of these QTLs revealed candidate genes involved in plant immunity (Kamfwa et al., 2015; Vidotti et al., 2019; Yassue et al., 2023), hormonal pathways (Vidotti et al., 2019; Cotta et al., 2020; Plucani do Amaral et al., 2023), nutrient uptake and provision (Stanton-Geddes et al., 2013; Curtin et al., 2017; Torkamaneh et al., 2020; Plucani do Amaral et al., 2023; Yassue et al., 2023) and plant development (Wintermans et al., 2016; Cotta et al., 2020), which is in line with the main pathways identified by analysis of mutants affecting microbiota structure in plants (Bergelson et al., 2021).

While informative, most of these GWAS were conducted with PGPB isolated from the belowground compartment of plants (*e.g.* roots and rhizosphere), thereby neglecting the close interplay between plant genetics and PGPB isolated from the phyllosphere (Copeland et al., 2015; Ahmed, 2017; Remus-Emsermann and Schlechter, 2018; Abadi et al., 2020; Chalot and Puschenreiter, 2021). In addition, the number of GWAS investigating the genetic architecture of plant response to native PGPB strains remains scarce, thereby impeding the discovery of genetic and molecular mechanisms that might have been selected during plant-PGPB co-evolution (Baltrus, 2017; Lyu et al., 2021). For instance, in the three GWAS conducted on *A. thaliana*, plants were inoculated with either the strain *Pseudomonas simiae* WCS417r isolated from the rhizosphere of wheat (Wintermans et al., 2016), the strain *Bacillus pumilus* TUAT-1 isolated from rice roots (Cotta et al., 2020) or the strain *Azoarcus oleearius* DQS-4^T^ isolated from oil-contaminated soil in Taiwan (Faoro et al., 2017; Plucani do Amaral et al., 2023). Finally, to our knowledge, it is still unknown whether plant polymorphic genes involved in interactions with PGPB have been shaped by natural selection. Yet, identifying candidate genes presenting suggestive signatures of local adaptation might be a starting point to unravel eco-evolutionary relevant biological pathways involved in the responsiveness of plants to PGPB (Bergelson and Roux, 2010; Roux and Bergelson, 2016).

In this study, we set up a GWAS under *in vitro* conditions to describe the genetic architecture of the response of *A. thaliana* to the bacterial species *Pseudomonas siliginis*. *P. siliginis* has been identified as the 6^th^ most abundant bacterial species in the leaf and root microbiota across 163 natural populations of *A. thaliana* located in the southwest of France (Bartoli et al., 2018). Based on a bacterial strain isolated from the rhizosphere of wheat, *P. siliginis* was recently described as a new species of the *Pseudomonas* genus (Girard et al., 2021). Since then, *P. siliginis* has been isolated from the phyllosphere of plants of the genus *Flaveria* (Murillo-Roos et al., 2022) and we recently isolated six strains of *P. siliginis* from the *A. thaliana* leaf compartment (Ramírez-Sánchez et al., 2022). Two and four of these strains showed a PGPB effect on *A. thaliana* when inoculated at the seed and seedling stages, respectively (Ramírez-Sánchez et al., 2022). By inoculating seeds of 162 whole-genome sequenced natural accessions from the southwest of France with one of these PGPB strains isolated in the same geographical region, we aimed at (i) estimating the extent of genetic variation of aboveground vegetative growth response to this strain at different time points, (ii) describing the underlying genetic architecture by combining a Bayesian hierarchical model with a local score approach that has been applied in diverse plant and animal species (Fariello et al., 2017; Bonhomme et al., 2019; Bonhomme et al., 2021; Aoun et al., 2020; Apuli et al., 2021; Libourel et al., 2021; Brachi et al., 2022; Demirjian et al., 2022; Andrews et al., 2023; Boitard et al., 2023; Demirjian et al., 2023; Frachon et al., 2023; Neto and Hancock, 2023; Roux et al., 2023), and (iii) evaluating the strength of selection acting on the candidate genes by testing whether the SNPs significantly associated with natural variation of the plant growth response to the strain overlapped significantly with suggestive signatures of local adaptation.

## Materials and methods

### Bacterial material

In this study, we used the OTU6_*Psi*_1 strain of *P. siliginis* that has been isolated from the rosette of one individual of *A. thaliana* collected in spring 2015 in the natural population ESPE-B located in the southwest of France (Bartoli et al., 2018; Ramírez-Sánchez et al., 2022). This strain was demonstrated to have a PGPB effect on *A. thaliana* under *in vitro* conditions when inoculated both at seed or seedling stage (Ramírez-Sánchez et al., 2022). In addition, we revealed a high genetic variation in response to this strain among seven *A. thaliana* accessions located in the southwest of France to be suppressed (Ramírez-Sánchez et al., 2022). Using single-molecule real-time long reads with a PacBio Sequel II system, a *de novo* genome sequence was obtained for the OTU6_*Psi*_1 strain, showing a single chromosome containing 5,458 genes (Ramírez-Sánchez et al., 2022).

### Plant material

Fifty-four populations (each represented by three accessions) were selected to represent the ecological and genetic diversity observed among a set of 168 natural populations of *A. thaliana* from southwest of France (Frachon et al., 2018; Frachon et al., 2019) (**Supplementary Table S1**). The seeds coming from the maternal plants were harvested in June 2015. To reduce differences in the maternal effects of the 162 seed lots (*i.e.* 54 populations × three accessions), one plant per accession per generation was grown as followed: (i) several seeds of each accession were sown on October 1^st^ 2016 in 7 x 7 x 6 cm plastic pots (Soparco^®^) filled with damp standard culture soil (PROVEEN MOTTE 20, Soprimex^®^); (ii) seeds were stratified at 4°C for four days; (iii) pots were put on November 4^th^ 2016 to a greenhouse at 22°C with a 16 hours photoperiod; (iv) seedlings were thinned to one on November 25^th^ 2016; (v) seedlings were transferred to the INRAE campus of Auzeville field station (France) on December 5^th^ 2016; (vi) when plants started to flower, they were moved to a greenhouse that reproduces outdoor conditions (no extra light or heating) but protects the plants from rain; (vii) aratubes (Arasystem^®^) were put on each plant to prevent cross-pollination between accessions; (viii) seeds were collected from late April to early May 2017 and conserved at 4°C.

### Seed sterilization

The sterilization of the seeds was performed with chlorine gas as previously described (Ramírez-Sánchez et al., 2022) and the seeds were then kept at 4°C.

### Experimental design and plant growth conditions

An experiment of 3,888 plants was set up using a split-plot design with two treatments (*i.e.* inoculation with OTU6_*Psi*_1 and mock treatment) nested within two blocks. Each ‘block × treatment’ interaction was represented by 24 48-well plates with each well filled with 700 µL of 0.5x MS medium (Murashige and Skoog medium), which contains 2.2 g of MS medium, 0.5 g of 2-(N-Morpholino)-ethanesulfonic acid, 6.0 g of plant tissue culture agar, 1 L of deionized water and a pH adjusted to the range of 5.7-5.8. The six wells of the last column of each plate were sown with seeds from the Col-0 reference accession to control for micro-environmental variation among plates. The 162 accessions were randomly assigned to the remaining columns of the 24 plates, resulting for each accession in a total of 12 replicates (two columns of six wells) in each treatment. The same randomization was done among treatments within a block but was modified between the two blocks.

For the 162 natural accessions and Col-0, one seed was sown in each well. After the 7-day cold treatment, seeds were inoculated and placed in a phytotron (10 hours photoperiod, light intensity ~ 80 µmol m^-2^ s^-1^, 21°C, 50% hygrometry) with a daily plate randomization.

### Inoculation

The OTU6_*Psi*_1 strain was grown from glycerol stock on solid medium in a 9 cm x 1.5 cm circle polystyrene Petri dish filled with TSA medium for one day. Colonies were diluted in 500 µl of sterile deionized water. 250 µl were then deposited in two new 9 cm x 1.5 cm circle polystyrene Petri dishes filled with TSA and spread with sterile beads. The two plates were incubated at 28°C overnight. Bacterial colonies were then resuspended and diluted in sterile deionized water to an OD_600nm_ of 0.5 which corresponds to 3.90 x10^8^ CFU/mL. Each seed was inoculated either with 5 µl of OTU6_*Psi*_1 (*P. siliginis* treatment) or 5 µl of sterile deionized water (mock treatment). Col-0 seeds that were sown in the last column of each 48-well plate were not inoculated. Plates were sealed with a micropore tape (3M Micropore Surgical x 9.14 m).

### Phenotyping

The germination date was recorded each day, between three and seven days after sowing. A picture of each 48-well plate was taken on 14, 21, and 28 days after inoculation (dai) using a photo-box designed in the lab and with a mobile camera (Samsung S6 16 Mpx). The vegetative growth was then visually scored for each plant using a scale established in Ramírez-Sánchez et al. (2022) and ranging from one (very small plant) to seven (well-grown plant). A total of 4,460 plants (3,888 plants of the 162 natural accessions and 572 Col-0 plants) were therefore phenotyped after inoculation at 14 dai, 21 dai and 28 dai.

### Statistical analyses

#### Natural genetic variation

We studied the genetic variation between the 54 natural populations of *A. thaliana* in response to OTU6_*Psi*_1 using the following mixed model (PROC MIXED procedure in SAS v. 9.4, SAS Institute Inc., Cary, NC, USA):


(Model 1) $$\begin{array}{l} Y_{ijklmn}=\mu_{trait}+Block_{i}+Treatment_{j}+Block_{i}*Treatment_{j}+Population_{k}+\\ Population_{k}*Treatment_{j}+Accession_{l}(Population_{k})+Accesion_{l}(Population_{k})*\\ Treatment_{j}+Germ_{m}+Score\_dai\_Col_{n}+\epsilon_{ijklmn} \end{array}$$

where Y corresponds to the score of plant development at a given dai; ‘µ’ is the overall mean of the phenotypic data; ‘Block’ accounts for differences in micro-environmental conditions between the two blocks; ‘Treatment’ corresponds to the mean effect of OTU6_*Psi*_1 in comparison with the mock treatment; ‘Population’ corresponds to the genetic differences among the 54 populations; ‘Accession(Population)’ corresponds to the mean genetic differences among accessions within populations; ‘Population*Treatment’ and ‘Accession(Population)*Treatment’ test if the rank among the 54 populations and the three accessions within populations differs among the two treatments, respectively; ‘Germ_date’ corresponds to the date of germination, ‘Score_dai_Col’ is a covariate that represents the mean value of the Col-0 plants for each plate and accounts for plate effects within a block; and ‘ϵ’ is the residual term. All factors were treated as fixed effects, as the levels of no factor were random samples from a population to which we intended to extrapolate. For calculating *F*-values, terms were tested over their appropriate denominators. As a split-plot design was set-up, the variance associated with ‘block × treatment’ was used as the error term to test ‘block’ and ‘treatment’ effects.

For each ‘treatment × dai’ combination, genotypic values of the 54 populations were estimated by calculating least-squares (LS) mean values of the term ‘Population’ in the following linear model (PROC MIXED procedure in SAS v. 9.4, SAS Institute Inc., Cary, NC, USA):


(Model 2) $$\begin{array}{l} Y_{ijklm}=\;{µ}_{trait}+Block_{i\;}+Population_{j}+Accession_{k}(Population_{j})+\\ Germ_{l}+Score\_dai\_Col_{m}+\;\epsilon_{ijklm}\; \end{array}$$

#### Broad-sense heritabilities

To calculate broad-sense heritability values (*H*²) of vegetative growth for each ‘treatment × dai’ combination, we first ran a linear model (PROC MIXED procedure in SAS v. 9.4, SAS Institute Inc., Cary, NC, USA):


(Model 3) $$Y_{ij}=\;{µ}_{trait}+Germ_{i}+Score\_dai\_Col_{j}+\;\epsilon_{ij}$$

We then ran the following model based on the residuals obtained from model 3:


(Model 4) $$Y_{ij}=\;{µ}_{trait}+Block_{i\;}+Accession_{j}+\;\epsilon_{ij}\;$$

The percentage of phenotypic variance explained by each term of Model 4 was estimated by the PROC VARCOMP procedure (REML method, SAS v. 9.4, SAS Institute Inc., Cary, NC, USA). *H*² values were then estimated as previously described (Lynch & Walsh, 1998; Huard-Chauveau et al., 2013) and using a formula adapted from Gallais (1990):


$$H_{Trait}^{2}\;=\;\frac{VF}{VF+\frac{VB}{B}+\frac{VR}{B\;*\;N}}$$

where ‘VF’ corresponds to the genetic variance among the 162 accessions, ‘*VB*’ is the variance associated with the ‘Block’ effect, ‘*B*’ is the number of blocks per treatment, ‘*VR*’ is the residual variance, and ‘*N*’ is the number of blocks.

At each dai, genotypic values of the 162 accessions were estimated by calculating LSmean values of the term ‘Accession’ of the following model (PROC MIXED procedure in SAS v. 9.4, SAS Institute Inc., Cary, NC, USA):


(Model 5) $$Y_{ijkl}=\;{µ}_{trait}+Block_{i\;}+Accession_{j}+Germ_{k}+Score\_dai\_Col_{l}+\;\epsilon_{ijkl}\;$$

#### Extent of plant growth response to OTU6_*Psi*_1 strain

Based on genotypic values, we estimated the extent of plant growth response (PGR) to OTU6_*Psi*_1 for each population and each accession at 14 dai, 21 dai and 28 dai using the following formula:


$$\text{PGR}\;=(\frac{\text{Genotypic value }(P.siliginis)-\text{Genotypic value }(\text{Mock})}{\text{Genotypic value }(\text{Mock})})*100$$

### Combining a Bayesian hierarchical model with a local score approach (BHM-LS)

Based on within-population genetic variation previously available for 168 natural populations of *A. thaliana* (Frachon et al., 2018), a Bayesian hierarchical model (Gautier, 2015) was applied to estimate the standardized allele frequencies corrected for the effect of population structure within each population for 1,638,649 SNPs across the genome (Frachon et al., 2018; Frachon et al., 2019). Standardized population allele frequencies were then retrieved for the 54 populations used in this work. Then, for each of the three PGR traits (*i.e.* PGR estimated at 14 dai, 21 dai and 28 dai), a genome scan was launched by estimating for each SNP the Spearman’s *rho* value and associated *p-*values between standardized allele frequencies and genotypic values obtained at the population level. Manhattan plots and quantile-quantile plots drawn on the *p*-values associated with Spearman’s *rho* values indicate an absence of an excess of low *p*-values. To better describe the genetic architecture associated with PGR, notably the identification of QTLs with small effects, we then implemented a local score approach on the set of *p*-values (Fariello et al., 2017). The local score allows detection of significant genomic segments by accumulating the statistical signals derived from multiple adjacent SNPs, thereby limiting the number of tests performed while utilizing all the available data (Fariello et al., 2017; Apuli et al., 2021). By following Bonhomme et al. (2019); Aoun et al. (2020); Libourel et al. (2021); Demirjian et al. (2022) and Demirjian et al. (2023), we then implemented a local score approach (with tuning parameter ξ = 2) on these *p* values. Finally, significant SNP-phenotype associations were found by estimating a chromosome-wide significance threshold for each chromosome (Bonhomme et al., 2019).

### Enrichment in biological processes

For each of the three PGR traits, the candidate genes underlying the QTLs were retrieved using a custom script written under the *R* environment (Libourel et al., 2021). The lists of the candidate genes were then submitted to the classification SuperViewer tool on the University of Toronto website (http://bar.utoronto.ca/ntools/cgibin/ntools_classification_superviewer.cgi) using the MapMan classification, to allow the identification of biological pathways significantly over-represented (*P<* 0.05).

### Enrichment in suggestive signatures of local adaptation

To test whether the SNPs underlying the QTLs identified by BHM-LS (hereafter named top SNPs) have suggestive signatures of local adaptation, we followed the method previously described in Brachi et al. (2015) for each of the three PGR traits. We looked for an over-representation of the top SNPs in the extreme upper tail of the XtX distribution obtained for the set of 168 natural populations of *A. thaliana* (Frachon et al., 2018). For a given SNP, XtX is a measure of the variance of the standardized population allele frequencies, which results from a rescaling based on the covariance matrix of population allele frequencies (Gautier, 2015). The formula used to calculate the fold enrichment in suggestive signatures of local adaptation was:


$${\text{FE}}_{\text{XtX}}=\frac{n_{a}/n}{N_{a}/N}$$

Here *n* is the number of SNPs in the upper tail of the XtX distribution. Here, we considered XtX statistic values as suggestive of local adaptation if they were among the top 1% of genome-wide XtX statistic values (*i.e.* 16,386 SNPs). *n_a_* is the number of top SNPs that were also in the upper tail of the XtX distribution. *N* is the total number of SNPs tested genome-wide and *Na* is the total number of top SNPs. Following the methodology described in Hancock et al. (2011), the statistical significance of enrichment was assessed by running 10,000 null circular permutations across the five chromosomes of *A. thaliana*.

## Results

**Figure 1:**
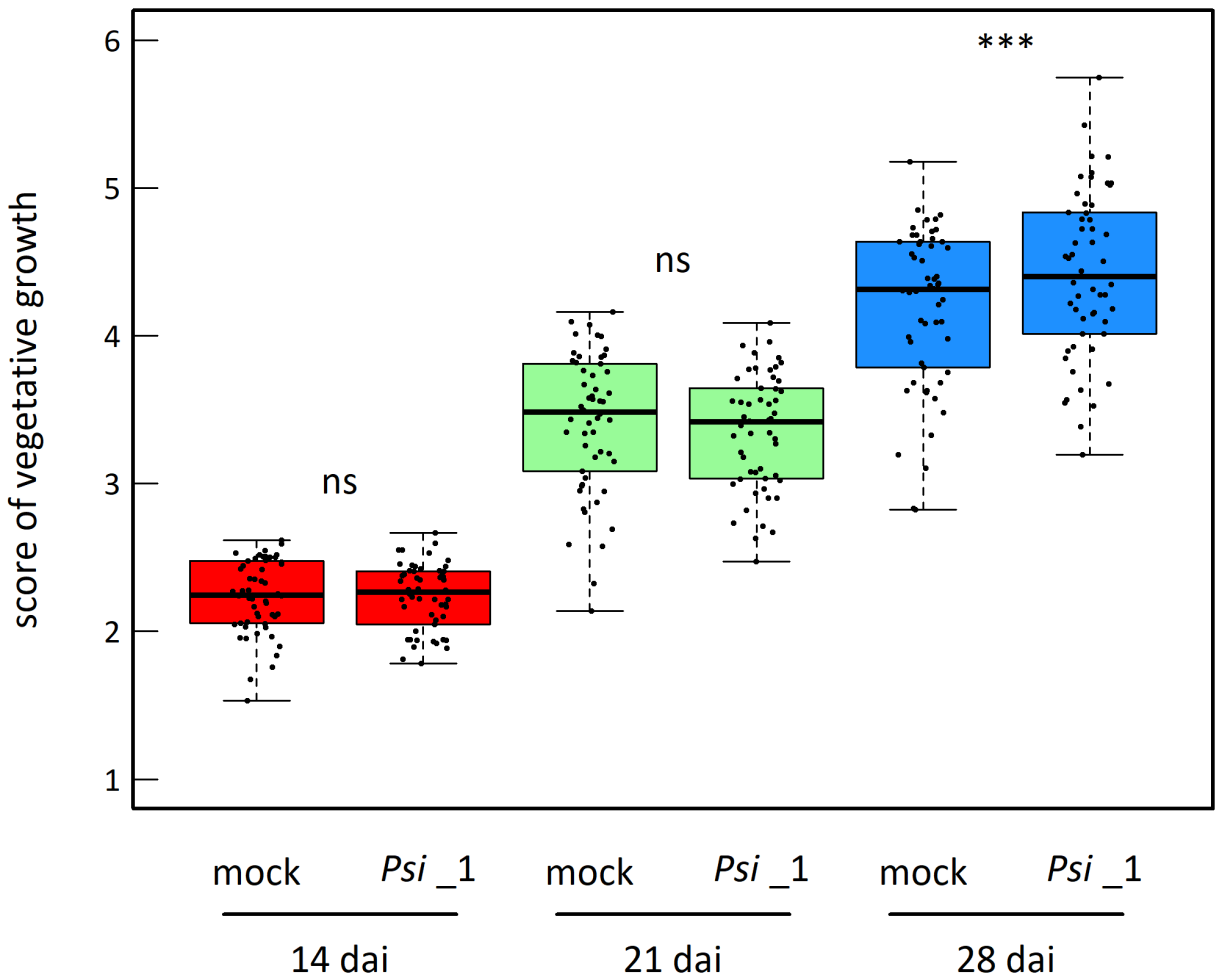
Mean genetic response to seed inoculation with the OTU6_*Psi*_1 strain at 14 dai, 21 dai and 28 dai. Each dot corresponds to the genotypic value of one of the 54 natural populations of *A. thaliana*. ns, non-significant, *** *P<* 0.001.

**Figure 2:**
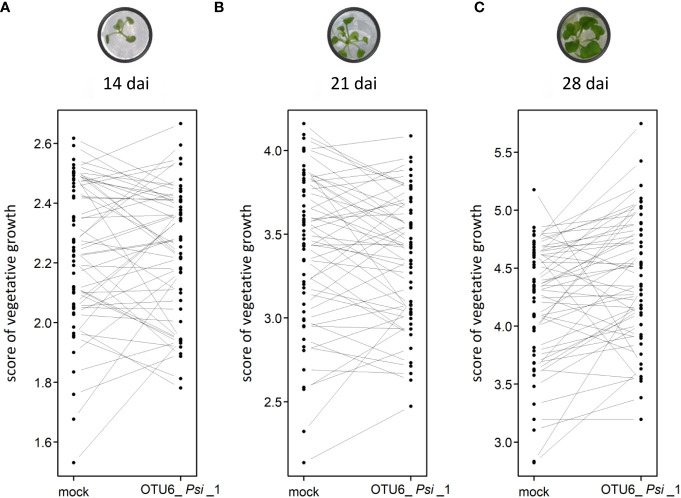
Interaction plots illustrating the genetic variation of response to the OTU6_*Psi*_1 strain at the population level at 14 dai **(A)**, 21 dai **(B)** and 28 dai **(C)**. Each dot corresponds to the genotypic value of one of the 54 populations of *A thaliana*. Each line corresponds to the response of one of the 54 populations to the inoculation with the OTU6_*Psi*_1 strain.

**Figure 3:**
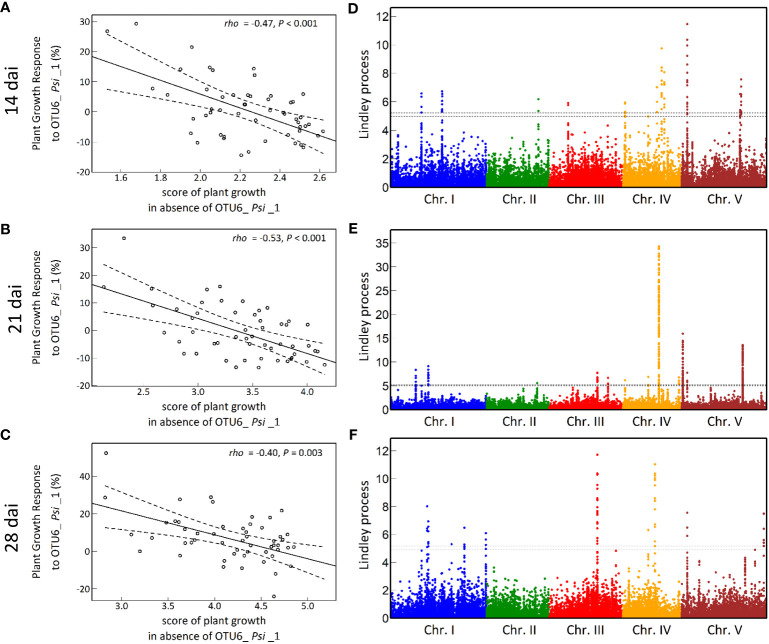
The genetic architecture of plant growth response (PGR) to the OTU6_*Psi*_1 strain. **(A-C)** Negative trade-offs at the population level between the level of PGR to OTU6_*Psi*_1 (expressed in percentage relative to the mock treatment) and the score of plant growth in absence of OTU6_*Psi*_1, at 14 dai, 21 dai and 28 dai. Each dot corresponds to the genotypic value of one of the 54 natural populations of *A. thaliana*. *rho*: correlation coefficient of Spearman between the response to OTU6_*Psi*_1 and the score of plant growth in absence of OTU6_*Psi*_1. *P*: *p*-value. The solid line corresponds to the fitted regression line, whereas the dashed lines delimit the band of 99% confidence intervals. **(D-F)** Manhattan plots of the Lindley process for PGR to OTU6_*Psi*_1 at 14 dai, 21 dai and 28 dai. The *x*-axis corresponds to the physical position of 1,638,649 SNPs on the five chromosomes. The dashed line indicates the chromosome-wide significance threshold.

The vegetative growth of the 54 natural populations of *A. thaliana* was on average significantly promoted by seed inoculation with the OTU6_*Psi*_1 strain of *P. siliginis* at 28 dai, but not at 14 dai and 21 dai (**Table 1**, **Figure 1**). At each time point of scoring, significant quantitative genetic variation was detected among populations as well as among accessions within populations across the two treatments (**Table 1**, **Figure 1**). Based on the 162 natural accessions, we detected significant and high broad-sense heritability (*H*²) values for each ‘treatment × time point of scoring’ combination (mock - 14 dai: *H*² = 0.80, *P<* 0.001; mock - 21 dai: *H*² = 0.79, *P<* 0.001; mock - 28 dai: *H*² = 0.82, *P<* 0.001; OTU6_*Psi*_1 - 14 dai: *H*² = 0.80, *P<* 0.001; OTU6_*Psi*_1 - 21 dai: *H*² = 0.80, *P<* 0.001; OTU6_*Psi*_1 - 28 dai: *H*² = 0.81, *P<* 0.001), suggesting that a large fraction of vegetative growth variation is explained by host genetic differences in our *in vitro* conditions. At each time point of scoring, we also detected a large and significant genetic variation among the 54 natural populations as well as among accessions within populations, for both the direction and the strength of the PGR to inoculation with the OTU6_*Psi*_1 strain (**Table 1**, **Figure 2**). Importantly, we observed a negative trade-off between the direction and the strength of the PGR to the OTU6_*Psi*_1 strain and the score of plant growth in absence of OTU6_*Psi*_1, with OTU6_*Psi*_1 having a positive effect on the vegetative growth of small plants and a negative effect on the vegetative growth of large plants (**Figures 3A-C**, **Supplementary Table S2**). This negative trade-off was observed at both the population and accession levels (**Figures 3A-C**, **Supplementary Figure S1**), thereby suggesting a phenomenon occurring among natural populations and within populations.

**Table 1:** Natural genetic variation of plant growth response to seed inoculation with the OTU6_*Psi*_1 strain at 14 dai, 21 dai and 28 dai.

Model terms	14 dai		21 dai		28 dai	
	*F*	*P*	*F*	*P*	*F*	*P*
Block	71.0	**1.04E-16**	14.7	1.81E-01	24.0	**1.81E-06**
Treatment	0.4	5.45E-01	0.7	5.46E-01	14.0	**3.19E-04**
Population	5.6	**1.00E-32**	6.0	**1.00E-32**	5.2	**2.53E-29**
Accession (Population)	4.6	**1.00E-32**	4.0	**1.00E-32**	3.5	**2.08E-26**
Treatment x Population	1.5	**1.98E-02**	1.2	1.50E-01	1.4	**4.94E-02**
Accession(Population) x Treatment	1.5	**2.93E-03**	1.3	**2.33E-02**	1.5	**2.75E-03**
Germination date	506.1	**1.00E-32**	581.7	**1.00E-32**	397.5	**1.00E-32**
Score Col-0 control	2.1	1.72E-01	0.4	5.45E-01	0.6	4.77E-01

Significant *p*-values after a False Discovery Rate correction are in bold.

**Figure 4:**
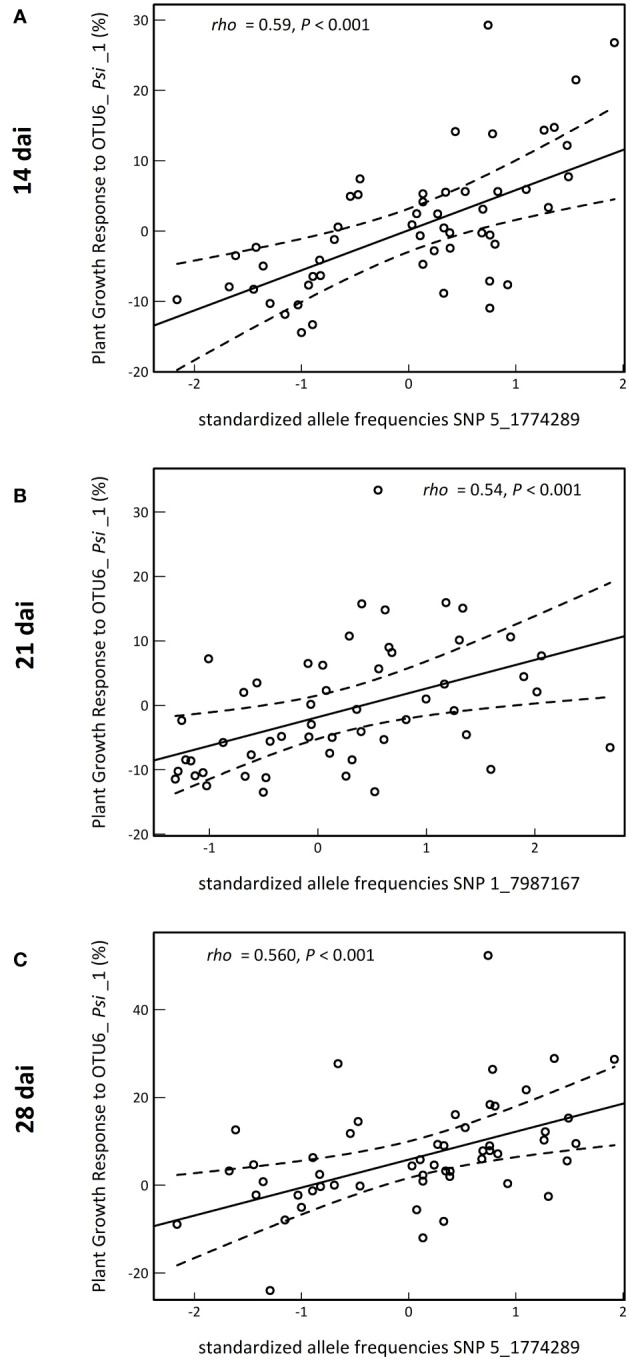
Illustration of the relationship between the level of plant growth response to the OTU6_*Psi*_1 strain (expressed in percent to the mock treatment) and the standardized allele frequencies of a top SNP presenting both one of the highest genotype-phenotype relationships and a signature of local adaptation at 14 dai **(A)**, 21 dai **(B)** and 28 dai **(C)**. *rho*: correlation coefficient of Spearman. *P*: *p*-value. The solid line corresponds to the fitted regression line, whereas the dashed lines delimit the band of 99% confidence intervals.

A GWA mapping analysis combining a Bayesian hierarchical model with a local score approach (BHM-LS) revealed a polygenic architecture of PGR to the OTU6_*Psi*_1 strain, with the identification of a total of 570 top SNPs underlying a total of 43 QTLs, with 14, 18 and 11 QTLs detected at 14 dai, 21 dai and 28 dai, respectively (**Figures 3D-F**; **Supplementary Tables S3**, **S4**). The genetic architecture was highly dynamic over time, with five QTLs in common between the three time points of scoring (**Figures 3D-F**, **Supplementary Table S4**). Importantly, the top SNPs were significantly enriched in suggestive signatures of local adaptation across the genome of *A. thaliana* in southwest of France, with a fold enrichment (FE) that increases with the time of scoring (14 dai: FE = 2.4, *P* = 0.0763; 21 dai: FE = 3.5, *P* = 0.0236; 28 dai: FE = 5.2, *P* = 0.0092) (**Supplementary Table S5**). Relationships between PGR variation and allele frequencies of a top SNP presenting a suggestive signature of local adaptation are illustrated for each time point of scoring in **Figure 4**.

**Figure 5:**
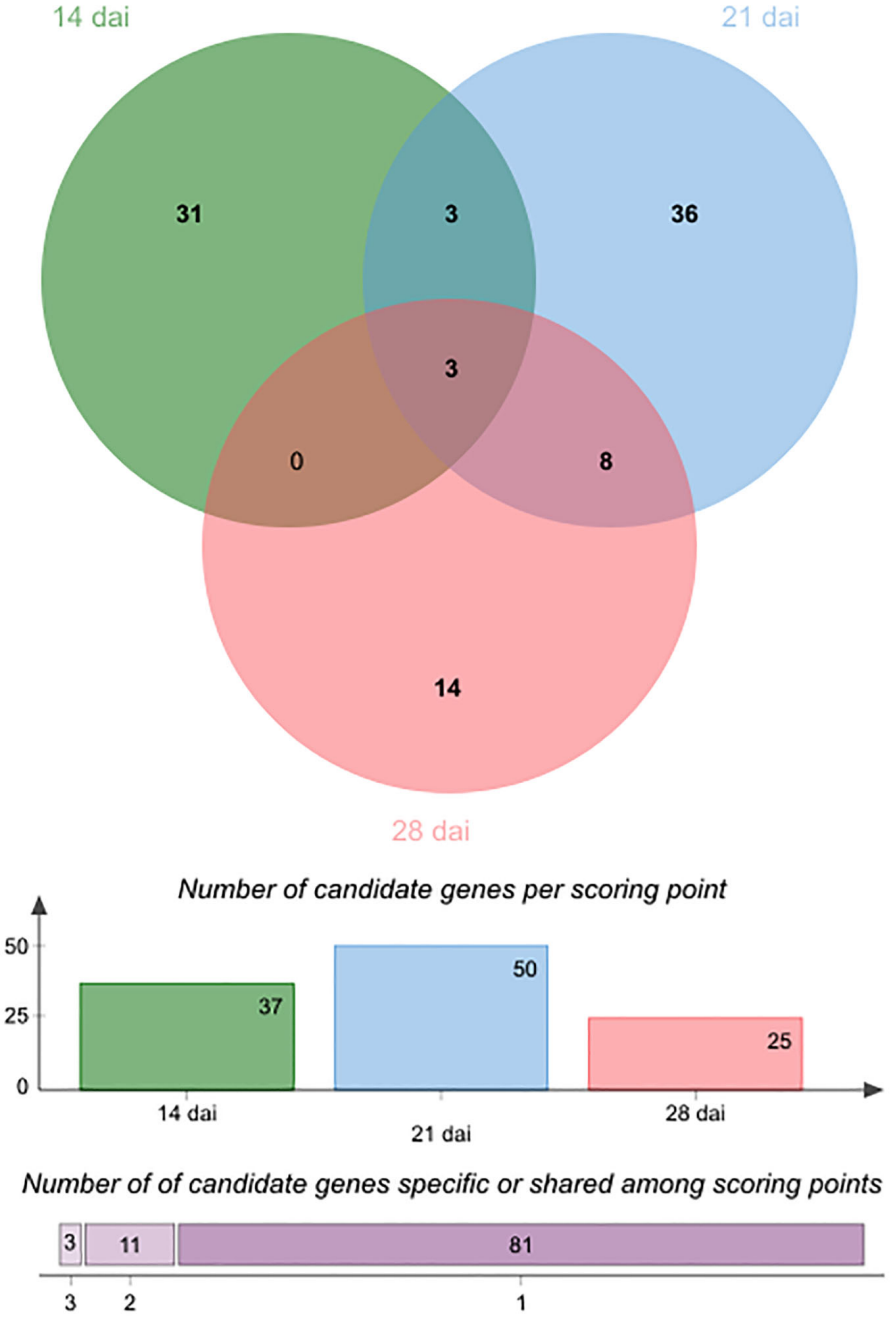
Venn diagram illustrating the number of specific and common candidate genes of plant growth response to the OTU6_*Psi*_1 strain between the three different time points of scoring. Colored bars indicate the number of candidate genes for each time point. The horizontally stacked bar plot indicates the number of candidate genes specific to one time point of scoring or common between two or three time points of scoring.

In line with the very short linkage disequilibrium of ~50 bp observed in French mapping populations of *A. thaliana* at the regional and local scales (Brachi et al., 2013; Frachon et al., 2017), the mean length of QTL intervals was rather small (~803 bp, quantile 5% ~ 47 bp, quantile 95% ~ 3.09 kb) (**Supplementary Table S4**), thereby allowing the fine mapping of candidate genes. Accordingly, the 43 detected QTLs overlapped with only 95 unique candidate genes, including 37, 50 and 25 unique candidate genes at 14 dai, 21 dai and 28 dai, respectively (**Supplementary Table S4**). In agreement with the dynamic genetic architecture between the three time points of scoring (**Figure 3**), only 14 candidate genes were common between two or three time points of scoring (**Table 2**, **Figure 5**). Interestingly, 4 out of the 14 candidate genes encode glycosyl transferases (GTs), including the three previously studied genes *AtGALT31A*, *UGT76C4*, and *UGT76C5* (Geshi et al., 2013; Li et al., 2015; Poulsen et al., 2015; Liu et al., 2019). *AtGALT31A*, which encodes a ß–galactosyltransferase involved in the elongation of ß–1,6-galactan side chains on arabinogalactan proteins, is important for the progression of embryo development beyond the globular stage (Geshi et al., 2013). UGT76C4 and UGT76C5 are two nicotinate N-glycosyltransferases (Li et al., 2015; Liu et al., 2019), with a proposed physiological function for UGT76C4 in seed development and germination. While *UGT76C4* is predominantly expressed in dry seeds, *UGT76C5* was mainly detected in the root tissue of 7-day-old seedlings (Li et al., 2015). We also identified the *AtCathB3* gene, encoding a cathepsin B-like protease, which is strongly induced during seed germination and early post-germination in *A. thaliana* (Iglesias-Fernández et al., 2014). Two other candidates, SBT3.5 and RLP48 (Receptor Like Protein 48), were previously characterized for their role in root growth and root hair development, respectively (Sénéchal et al., 2014; Stetter et al., 2015). The subtilisin-like serine protease SBT3.5 may play a role in the regulation of *PME17* encoding a putative pectin methylesterase (PME) in *A. thaliana* roots (Sénéchal et al., 2014). First identified as a candidate gene in a GWAS on root hair traits in response to the scarce local phosphorus supply, RLP48 was then validated as being involved in root hair density (Stetter et al., 2015). Finally, an interesting candidate is *JAZ11*, a gene part of the jasmonate (JA)-zinc-finger inflorescence meristem (ZIM)-domain (JAZ) family (Liu et al., 2021). The *jaz11* mutant exhibits JA-regulated root growth inhibition and increased susceptibility to *Pseudomonas syringae* pv. *tomato* (*Pst*) DC3000 (Liu et al., 2021).

**Table 2:** List of the 14 candidate genes in response to the OTU6_*Psi*_1 strain and common between two or three time points of scoring.

ATG number	Common time points	Subcategory	Function
*At1g32928*	21-28 dai	not assigned unknown	
*At1g32930*	21-28 dai	protein glycosylation	AtGALT31A GALT31A Galactosyltransferase family protein
*At1g32940*	21-28 dai	protein degradation subtilase	AtSBT3.5 SBT3.5 Subtilase family protein
*At3g43440*	21-28 dai	not assigned unknown	JAZ11 TIFY3A jasmonate-zim-domain protein 11
*At3g43470*	21-28 dai	not assigned unknown	zinc ion binding, nucleic acid binding
*At4g01593*	14-21 dai	micro RNA, natural antisense etc	other RNA
*At4g01600*	14-21 dai	hormone metabolism abscisic acid induced-regulated-responsive-activated	GRAM domain family protein
*At4g01610*	14-21 dai	protein degradation cysteine protease	AtcathB3 Cysteine proteinases superfamily protein
*At4g13860*	21-28 dai	RNA RNA binding	RNA-binding (RRM/RBD/RNP motifs) family protein
*At4g13870*	21-28 dai	DNA unspecified	ATWEX ATWRNEXO WEX WRNEXO Werner syndrome-like exonuclease
*At4g13880*	21-28 dai	stress biotic	AtRLP48 RLP48 receptor like protein 48
*At5g05880*	14-21-28 dai	misc UDP glucosyl and glucoronyl transferase	UGT76C4 UDP-Glycosyltransferase superfamily protein
*At5g05890*	14-21-28 dai	misc UDP glucosyl and glucoronyl transferase	UGT76C5 UDP-Glycosyltransferase superfamily protein
*At5g05900*	14-21-28 dai	misc UDP glucosyl and glucoronyl transferase	UDP-Glycosyltransferase superfamily protein

We also looked for biological processes significantly over-represented compared to the overall class frequency in the *A. thaliana* MapMan annotation. This allowed us to identify relevant candidate genes of PGR to the OTU6_*Psi*_1 strain. Based on the lists of unique candidate genes found for each time point of scoring, we detected one and two significantly over-represented biological processes at 14 dai and 21 dai, respectively. The over-represented biological process at 14 dai corresponds to the ‘cell wall modification’ class, whereas the over-represented biological processes at 21 dai correspond to the ‘co-factor and vitamin metabolism’ and ‘nucleotide metabolism’ classes. No significantly over-represented biological processes were detected at 28 dai. The three enriched classes contain seven candidate genes (**Supplementary Table S4**). For the ‘cell wall modification’ class, *EXP17* encodes an expansin, a non-hydrolytic cell wall-loosening protein, which was suggested to participate in cell separation to promote lateral roots (LRs) emergence via the overlaying tissues of the primary root. Overexpression and silencing of *EXP17* in *A. thaliana* increased and delayed the density of emerged LRs in the presence of auxin, respectively (Lee and Kim, 2013). The two other cell wall remodeling genes encode the xyloglucan endotransglucosylases/hydrolases *XTH14* and *XTH23* (Maris et al., 2009; Xu et al., 2020). *XTH23* is involved in LR development under salt stress (Xu et al., 2020). The ‘co-factor and vitamin metabolism’ class contains two genes. *Pyridoxine synthase 1* (*PDX1.1*) is part of a specific pathway involved in the biosynthesis of vitamin B_6_ (pyridoxal 5’-phosphate) in higher plants, which acts as a coenzyme for many metabolic enzymes but also as a potent antioxidant (Tambasco-Studart et al., 2005). Strikingly, *pdx1* knockout mutants are impaired in root growth and early seedling development and are hypersensitive to osmotic and oxidative stresses (Chen and Xiong, 2005; Boycheva et al., 2015). *AtFMN/FHy* encodes a bi-functional enzyme involved in the metabolism of vitamin B_2_ (riboflavin) (Maruta et al., 2012). The ‘nucleotide metabolism’ class also contains two genes. *AtNUDX2* is part of an *A. thaliana* Nudix (nucleoside diphosphates linked to some moiety X) hydrolase family of 28 genes. *AtNUDX2* encodes an ADP-ribose pyrophosphatase that confers enhanced tolerance of oxidative stress in *A. thaliana* (Ogawa et al., 2009; Maruta et al., 2012). *AMK2* encodes an adenosine monophosphate kinase that has a role in the architecture of chloroplasts (Lange et al., 2008).

## Discussion

### High genetic variation of plant growth response to a native PGPB at a regional scale

Despite the phyllosphere representing 60% of the total biomass on Earth and concentrating 10^26^ bacteria (Vorholt, 2012), most GWAS carried out with non-pathogenic bacteria have focused on symbiotic bacteria or non-symbiotic PGPB isolated from the belowground compartment of plants (Stanton-Geddes et al., 2013; Wintermans et al., 2016; Curtin et al., 2017; Vidotti et al., 2019; Cotta et al., 2020; Torkamaneh et al., 2020). In this study, extensive genetic variation was observed among 162 natural accessions of *A. thaliana* in response to one strain of the native PGPB *P. siliginis*, which is an abundant and prevalent bacterial species in the leaf and root compartments of natural populations of *A. thaliana* located in the southwest of France (Ramírez-Sánchez et al., 2022). Because other strains of *P. siliginis* have been isolated from the leaf compartment of *A. thaliana* and characterized at the genomic level (Ramírez-Sánchez et al., 2022), it would be interesting to test whether the level of genetic variation of PGR and the underlying genetic architecture are similar among *P. siliginis* strains when inoculated at the seed stage. In addition, because *P. siliginis* and other *Pseudomonas* species with a PGPB effect, such as *Pseudomonas moraviensis* (Ramírez-Sánchez et al., 2022), belong to the subgroup *Pseudomonas koreensis* (Girard et al., 2021), it would be informative to check whether the genetics of PGR to *P. siliginis* extends to other phylogenetically close *Pseudomonas* species. Finally, in agreement with seed coating as an efficient way of introducing PGPB to seedlings (Ma, 2019; de Souza et al., 2020), we set up our GWAS by inoculating *P. siliginis* on seeds. Because the strength of the PGPB effect of *P. siliginis* on *A. thaliana* can depend on the developmental stage of the plants (Ramírez-Sánchez et al., 2022), it would be complementary to set up a GWAS by inoculating *P. siliginis* at the seedling stage.

Importantly, we observed a strong negative trade-off between plant growth in absence of *P. siliginis* and PGR to the OTU6_*Psi*_1 strain. To our knowledge, such a negative trade-off has not been reported in the literature. Identifying the mechanisms underlying this negative trade-off deserves further investigation. For instance, these mechanisms might rely on differences in seed size and physiology among the 162 accessions tested in this study. Beyond identifying the mechanisms, such a negative trade-off should promote the maintenance of genetic diversity at the underlying candidate genes, with the selection of growth-inductor responsive *A. thaliana* genotypes in presence of *P. siliginis* and the selection of growth-inhibitor responsive *A. thaliana* genotypes in absence of *P. siliginis*. Because the trade-off was observed both at the among-population and within-population levels, it suggests that the dynamics of *A. thaliana* - PGPB interactions should be studied at the metapopulation level rather than at the population level, as previously evidenced by studies on natural plant pathosystems such as *Plantago lanceolata* -*Podosphaera plantaginis* (Laine, 2005; Thrall et al., 2012) and *A. thaliana* - *Pseudomonas syringae* (Karasov et al., 2014).

### The genetic architecture of response to a native PGPB is dynamic and potentially adaptive

Theoretical predictions suggest that the temporal regulation of QTLs often drives phenotypic changes in ontogenetic time, typically time-to-event or time-to-failure traits such as flowering time or death time (Johannes, 2007). Accordingly, previous GWAS performed on plant response to pathogens revealed temporal patterns in the detection of QTLs along the infection stages, with association peaks being detected only either at the earlier or at the later stages of infection (Aoun et al., 2017; Bartoli and Roux, 2017; Aoun et al., 2020; Demirjian et al., 2022; Demirjian et al., 2023). For instance, the atypical meiotic cyclin *SOLO DANCERS* gene was functionally validated in *A. thaliana* as conferring susceptibility to the bacterial pathogen *Ralstonia solanacearum* but only at the early stages of the infection (Aoun et al., 2020). Another *A. thaliana* gene, *BWS1* (*bacterial wilt susceptibility 1*), was also revealed by GWAS as a susceptibility factor with a temporal dynamic in response to *R. solanacearum* (Demirjian et al., 2023). In this study, a similar dynamic in the detection of QTLs was observed for PGR to the OTU6_*Psi*_1 strain, suggesting that the PGPB effects conferred by *P. siliginis* depend on the time specificity of the genetic effects of *A. thaliana.* Importantly, in line with the mean PGPB effect of the strain OTU6_*Psi*_1 that increases over time, the enrichment in suggestive signatures of local adaptation of the candidate genes also increases over time, thereby highlighting the eco-evolutionary relevance of this native *A. thaliana* -* P. siliginis* interactions, similarly to the native interactions between *A. thaliana* and the bacterial pathogen *P. syringae* (Karasov et al., 2014; Roux and Bergelson, 2016). Our population genomics approach for identifying suggestive signatures of local adaptation across the genome of *A. thaliana* allows taking into account both the effect of selective processes at all life stages of *A. thaliana* while controlling for the effect of local demographic history (Frachon et al., 2017; Roux et al., 2023). This indirect approach is based on the calculation of the XtX statistics, analogous to *F*_ST_ but explicitly corrected for the covariance matrix of allele frequencies among populations (Gautier, 2015), and has been used to identify candidate genes associated with suggestive signatures of local adaptation in diverse species such as the European white oak (Leroy et al., 2020), the Aleppo pine *Pinus halepensis* (Ruiz Daniels et al., 2019), *Caenorhabditis elegans* (Crombie et al., 2022), the white-footed mice *Peromyscus leucopus* (Harris and Munshi-South, 2017) and the fungal wheat pathogen *Zymoseptoria tritici* (Hartmann et al., 2018). The PGPB effect observed on vegetative growth should therefore translate to fitness proxies such as total seed production in natural conditions, but remains to be tested. However, although a direct approach for testing local adaptation is based on setting up field experiments, in particular reciprocal field experiments, some fitness components such as germination rate can be hard to estimate under natural conditions (Savolainen et al., 2013). In addition, we must caution that estimating the effect of an adaptive allele on fitness proxies such as total seed production does not always predict the fate of the evolution of the frequency of this allele, as previously demonstrated for alleles conferring herbicide resistance (Roux et al., 2004; Roux et al., 2005; Roux et al., 2006; Vila-Aiub et al., 2011).

### *P. siliginis* OTU6_*Psi*_1 might target genes involved in seed and root development kinetics to promote plant growth

Of the 21 candidate genes highlighted, *i.e.* 14 genes in common between two or three scoring time points and seven genes of the three enriched biological processes, more than half of them are potentially involved in cell wall proliferation during seed and root development. Among these candidates, we observed both primary (*i.e.* expansins) and secondary (*i.e.* endoglycosylase/hydrolases) wall-loosening factors that are key players in cell wall structuring. For instance, expansins are cell wall-loosening proteins that directly induce cell wall extension by breaking non-covalent bonds between cellulose micro-fibrils and associated matrix polysaccharides in the cell wall (Lee and Kim, 2013). This study highlights EXP17 and xyloglucan endotransglucosylases/hydrolases, which facilitate LR emergence (Maris et al., 2009; Lee and Kim, 2013; Xu et al., 2020). XTH isoenzymes also strengthen the side-walls and cell walls of root hairs in the root differentiation zone after the completion of cell expansion (Maris et al., 2009). In addition, we identified GTs that catalyze protein glycosylation, a major post-translational modification of proteins, which significantly affects protein folding, conformation, distribution, stability and activity (Poulsen et al., 2015). Specifically, we identified a galactosyltransferase (Geshi et al., 2013) and three UGTs (UDP-glycosyltransferases), which are described to glycosylate various phytohormones and metabolites in response to biotic and abiotic stress in plants (Li et al., 2015; Rehman et al., 2018). Finally, studying PDX1 revealed that vitamin B_6_ is essential for root development and stress tolerance (Chen and Xiong, 2005; Boycheva et al., 2015). Other candidates are involved in both growth development and plant defense. For instance, the candidate gene *SBT3.5* may have a direct or indirect role in root and/or root hair development, particularly *via* the processing of PME7 *in planta* (Sénéchal et al., 2014), as PME are ubiquitous cell wall enzymes involved in important developmental processes (Micheli, 2001). Beyond the role of SBT3.5 in root development, another subtilase, SBT3.3, plays a role in immune priming during plant-pathogen interactions (Ramírez et al., 2013). In addition, the candidate gene *JAZ11* inhibits *A. thaliana* hypersensitivity to the key phytohormone JA and represses susceptibility to *Pst* DC3000 (Liu et al., 2021).

The next step to understand the genetic and molecular mechanisms underlying the adaptive negative trade-off in response to *P. siliginis* observed in this study would be to phenotype (i) the mutant lines of the candidate genes for PGR of both leaves and roots, and (ii) the ability of the OTU6_*Psi*_1 strain to multiply in the leaf and root compartment of seedlings. In addition, it would be of particular interest to study the expression profiles with the spatial and subcellular localization of the candidate genes after inoculation with the OTU6_*Psi*_1 strain. Finally, exploiting the haplotypic diversity of the candidate genes among the 162 natural accessions of *A. thaliana* used in this study may help to identify the polymorphisms that have been selected in nature to respond to the PGPB *P. siliginis*.

## Data availability statement

The raw data supporting the conclusions of this article will be made available by the authors, without undue reservation.

## Author contributions

DR-S: Conceptualization, Formal Analysis, Funding acquisition, Investigation, Methodology, Validation, Writing – original draft, Writing – review & editing, Data curation, Software, Visualization. CG-V: Data curation, Formal Analysis, Investigation, Methodology, Validation, Writing – original draft, Writing – review & editing. FR: Data curation, Formal Analysis, Investigation, Methodology, Validation, Writing – original draft, Writing – review & editing, Conceptualization, Funding acquisition, Project administration, Resources, Software, Supervision, Visualization. FV: Conceptualization, Formal Analysis, Funding acquisition, Investigation, Methodology, Project administration, Resources, Supervision, Validation, Writing – original draft, Writing – review & editing.
