# Supplementary material for: Genetic architecture of the response of Arabidopsis thaliana to a native plant-growth-promoting bacterial strain
Source: Front Plant Sci. 2023 Nov 9;14:1266032. doi: 10.3389/fpls.2023.1266032 (PMC10665851; doi:10.3389/fpls.2023.1266032)
Supplement: Supplementary file 2 [file Presentation_1.pdf]

Supplementary Information

Genetic architecture of the response of *Arabidopsis thaliana* to a native plant-growth-promoting bacterial strain

Daniela Ramírez-Sánchez, Chrystel Gibelin-Viala, Fabrice Roux* and Fabienne Vailleau*

LIPME, INRAE, CNRS, Université de Toulouse, Castanet-Tolosan, France

* The authors share senior authorship.

* Corresponding author: fabienne.vailleau@inrae.fr (F. Vailleau)

This file includes:

Supplementary Figures 1-3

Supplementary Tables 1 and 5

(For Supplementary Tables 2-4 and Data set 1, see the Excel file)

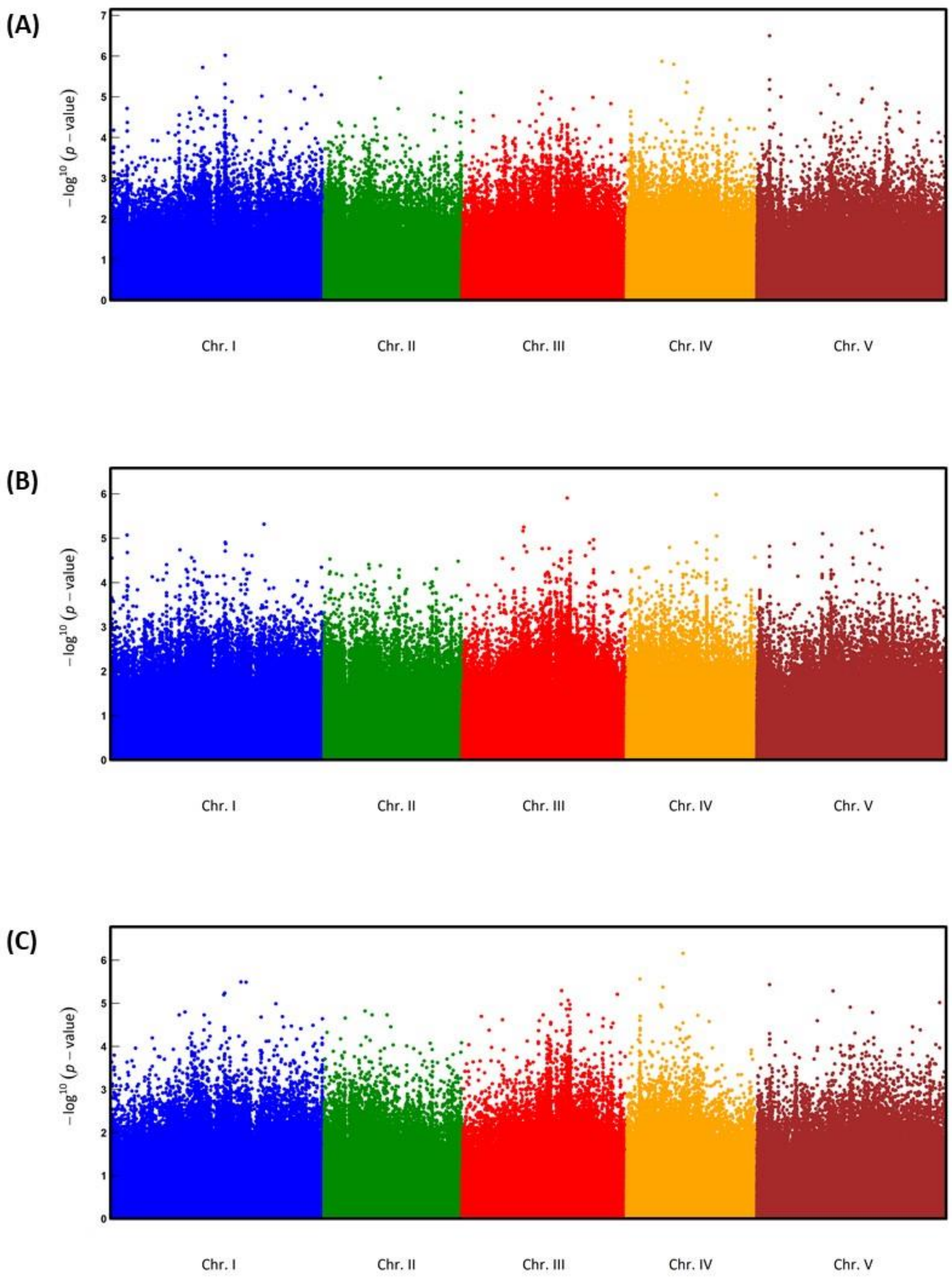

SUPPLEMENTARY FIGURE S1 | Manhattan plots of mapping results or PGR to OTU6_*Psi*_1 at 14 dai **(A)**, 21 dai **(B)** and 28 dai **(C)**. The *x*-axis corresponds to the physical position of 1,638,649 SNPs on the five chromosomes. The *y*-axis indicates the $-\log_{10}(P)$ of the Spearman's *rho* estimates.

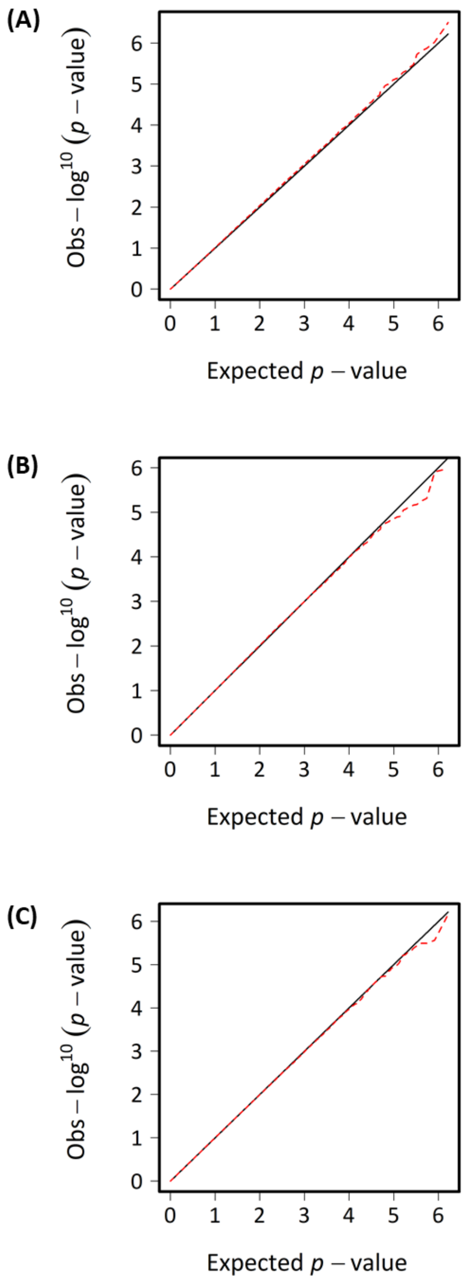

SUPPLEMENTARY FIGURE S2 | Quantile-quantile plot of $-\log_{10}$ *p*-values of the Spearman's *rho* estimates at 14 dai **(A)**, 21 dai **(B)** and 28 dai **(C).** Solid black line: expected distribution. Dotted red line: observed distribution.

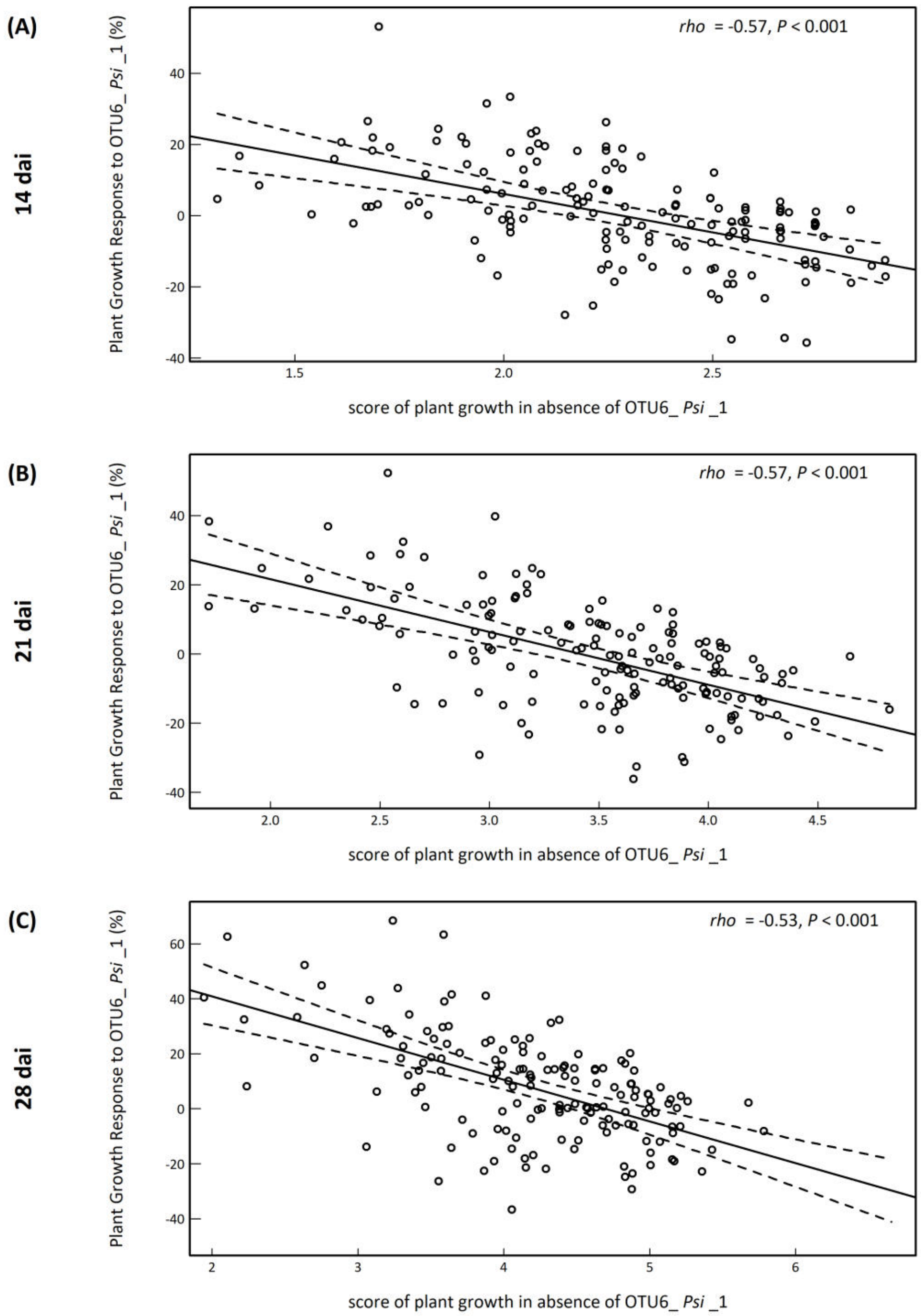

SUPPLEMENTARY FIGURE S3 | Illustration of the negative trade-offs at the accession level between the level of plant growth response (PGR) to the OTU6_*Psi*_1 strain (expressed in percentage relative to the mock treatment) and the score of plant growth in absence of seed inoculation with OTU6_*Psi*_1, at 14 dai (A), 21 dai (B) and 28 dai (C). Each dot corresponds to the genotypic value of one of the 162 natural accessions of *A. thaliana*. *rho*: correlation coefficient of Spearman between the response to OTU6_*Psi*_1 and the score of plant growth in absence of OTU6_*Psi*_1. *P*: *p*-value. The solid line corresponds to the fitted regression line, whereas the dashed lines delimit the band of 99% confidence intervals.

SUPPLEMENTARY TABLE S1 | Names and GPS coordinates (expressed in degrees) of the 54 populations used in this study.

Population name	Locality	Latitude	Longitude
ANGE-B	Saint Angel, Salvagnac	43.91214	1.656855
AULO-A	Aulon	43.190552	0.815774
BANI-B	Banios	43.043644	0.234303
BARA-B	Baraqueville	44.269727	2.426322
BELC-C	Belcastel	44.389212	2.336636
CAMA-C	Camarès	43.824878	2.881661
CASS-A	Cassagne-Begontes	44.17653	2.518164
CAST-A	Castelginset	43.698534	1.427856
CAZA-B	Cazaux-Fréchet	42.831484	0.420091
CERN-A	Saint-Rome-de-Cernon	44.01194	2.966488
CHEI-A	Chein-dessus	43.013708	0.86707
CIER-A	Cier sur Luchon	42.85332	0.602039
CIER-B	Cier de Luchon	42.859978	0.600413
CLAR-A	Saint Clar-de-Rivière	43.464776	1.219019
CLAR-B	Saint Clar-de-Rivière	43.465281	1.218577
CLAR-C	Saint Clar-de-Rivière	43.464058	1.21799
COLO-A	Colombiès	44.346915	2.340243
COLO-B	Colombiès	44.34773	2.339715
DECA-A	Châteaude Cas (Espinas)	44.199896	1.77189
DIEU-A	Ville-Dieu-du-temple	44.059797	1.220975
ESPE-B	Esperausses	43.693335	2.534582
FAYA-A	Fayet	43.8021	2.951709
FERR-A	Ferrières	43.657743	2.44371
GAIL-A	Gaillac	43.908928	1.900574
LABA-B	Labarthe-sur-Lèze	43.450892	1.40116
LABA-C	Labarthe-sur-Lèze	43.451451	1.39935
LABA-D	Labarthe-sur-Lèze	43.458019	1.381137
LABAS-B	La bastide de Sérou	43.008716	1.420053
LACR-A	Lacraste (Montgauch)	42.999869	1.075659
LACR-C	Lacraste (Montgauch)	43.000155	1.075624
LAGR-A	Lagraulhet St Nicolas	43.795323	1.073752
LAMA-B	Lamasquère	43.479745	1.241592
LANT-C	Lanta	43.564822	1.65201
LUZE-B	Luzenac (Garanou)	42.764419	1.753595
MAZA-A	Mazamet	43.497754	2.375372
MERE-A	Merens-les-Vals	42.656618	1.836221
MERV-A	Merville	43.720426	1.296824
MERV-B	Merville	43.725141	1.247629
MONB-A	Monblanc	43.46529	0.986273
MONF-A	Monferran-Savès	43.616254	0.972435
MONT-A	Montans	43.852212	1.87432
MONTI-A	Montiès	43.389383	0.67282
MONTI-B	Montiès	43.3839336	0.67257
MONTM-A	Montmajou (Cier de Luchon)	42.86156	0.595943
MONTM-B	Montmajou (Cier de Luchon)	42.861218	0.596869
MOUL-A	Moularès	44.089762	2.296094
NAUV-B	Nauviale	44.520418	2.427129
NAYR-A	Le Nayrac (Cassagnes-Bégontes)	44.161368	2.544711
SAMA-A	Samatan	43.494325	0.92391
SAUB-C	Saubens	43.475583	1.367589
SAUR-A	Saurat	42.889844	1.485209
SEIS-A	Seissan	43.487302	0.588798
TARN-C	Villemur-sur-Tarn	43.85328	1.502009
VALE-A	Valence d'Albiegeois	44.022296	2.403434

SUPPLEMENTARAY TABLE S5 | Top SNPs associated with natural variation of plant growth response (PGR) to the OTU6_*Psi*_1 strain and presenting suggestive signatures of local adaptation when considering the 1 % upper tail of the XtX distribution. rho: Spearman's correlation coefficient between the level of PGR to OTU6_*Psi*_1 and the standardized allele frequencies of a given top SNP. p-value: level of significance of the rho values.

trait	chromosome	position	SNP_ID	QTL ID	rho	p-value	Lindley	XtX value
Response_14dai	5	1774091	5_1774091	QTL11	0.384	0.00443793	7.51	191.95
Response_14dai	5	1774283	5_1774283	QTL11	0.400	0.00295674	8.04	194.68
Response_14dai	5	1774289	5_1774289	QTL11	0.592	3.82E-06	11.46	199.45
Response_21dai	1	11932723	1_11932723	QTL2	-0.356	0.0085815	7.40	192.50
Response_21dai	1	7986738	1_7986738	QTL1	0.428	0.00137103	0.86	192.82
Response_21dai	1	7987167	1_7987167	QTL1	0.536	3.96E-05	4.90	199.52
Response_21dai	4	11428185	4_11428185	QTL10	-0.456	0.00060164	2.82	195.19
Response_21dai	4	11429168	4_11429168	QTL10	-0.406	0.00252046	3.85	201.90
Response_21dai	4	11430404	4_11430404	QTL10	0.089	0.52181908	9.93	197.46
Response_21dai	4	11430678	4_11430678	QTL10	0.209	0.13001488	8.81	191.75
Response_21dai	4	11433485	4_11433485	QTL10	-0.378	0.00513639	27.02	196.39
Response_21dai	5	19313854	5_19313854	QTL15	-0.402	0.00274724	2.70	212.29
Response_21dai	5	19313916	5_19313916	QTL15	-0.460	0.0005404	4.10	197.81
Response_21dai	5	420494	5_420494	QTL12	-0.118	0.39416093	5.07	200.72
Response_21dai	5	422548	5_422548	QTL13	0.396	0.00325604	14.16	192.57
Response_28dai	1	11932723	1_11932723	QTL2	-0.360	0.00777792	5.45	192.50
Response_28dai	4	8024318	4_8024318	QTL8	-0.390	0.0037375	0.43	199.58
Response_28dai	5	1774091	5_1774091	QTL10	0.366	0.00685426	3.70	191.95
Response_28dai	5	1774283	5_1774283	QTL10	0.390	0.00376641	4.13	194.68
Response_28dai	5	1774289	5_1774289	QTL10	0.593	3.70E-06	7.56	199.45
